# Genetic structuring of the coastal herb *Arthropodium cirratum* (Asparagaceae) is shaped by low gene flow, hybridization and prehistoric translocation

**DOI:** 10.1371/journal.pone.0204943

**Published:** 2018-10-17

**Authors:** Lara D. Shepherd, Mariana Bulgarella, Peter J. de Lange

**Affiliations:** 1 Museum of New Zealand Te Papa Tongarewa, Wellington, New Zealand; 2 Environment and Animal Sciences, Unitec Institute of Technology, Auckland, New Zealand; National Cheng Kung University, TAIWAN

## Abstract

We examined the genetic structuring of rengarenga (*Arthropodium cirratum*; Asparagaceae), an endemic New Zealand coastal herb, using nuclear microsatellite markers. This species was brought into cultivation by Māori within the last 700–800 years for its edible roots and was transplanted beyond its natural distribution as part of its cultivation. We found very high levels of genetic structuring in the natural populations (F_ST_ = 0.84), indicating low levels of gene flow. Reduced genetic diversity was found in the translocated populations, suggesting a large loss of genetic diversity early in the domestication process. The data indicates that rengarenga was brought into cultivation independently at least three times, with the sources of these introductions located within a narrow area encompassing about 250km of coastline. Hybridization was inferred between *A*. *cirratum* and the closely related *A*. *bifurcatum*, despite *A*. *birfucatum* not occurring in the vicinity.

## Introduction

New Zealand was the last substantial landmass to be colonised [1], about 700–800 years ago [2]. Pacific Islanders translocated and cultivated many food, medicinal and fibre plants as they colonised the Pacific [3]. Six of these were known to be cultivated in New Zealand by Māori at the time of European contact but their cultivation was marginal owing to New Zealand’s relatively cooler climate. Instead Māori began to cultivate several endemic New Zealand plant species for food, fibre and medicine [4]. These cultivated endemic plants are useful for studying the domestication process because their initial cultivation must have been no later than 800 years ago, and they therefore provide a window into the early stages of the domestication process.

**Fig 1:**
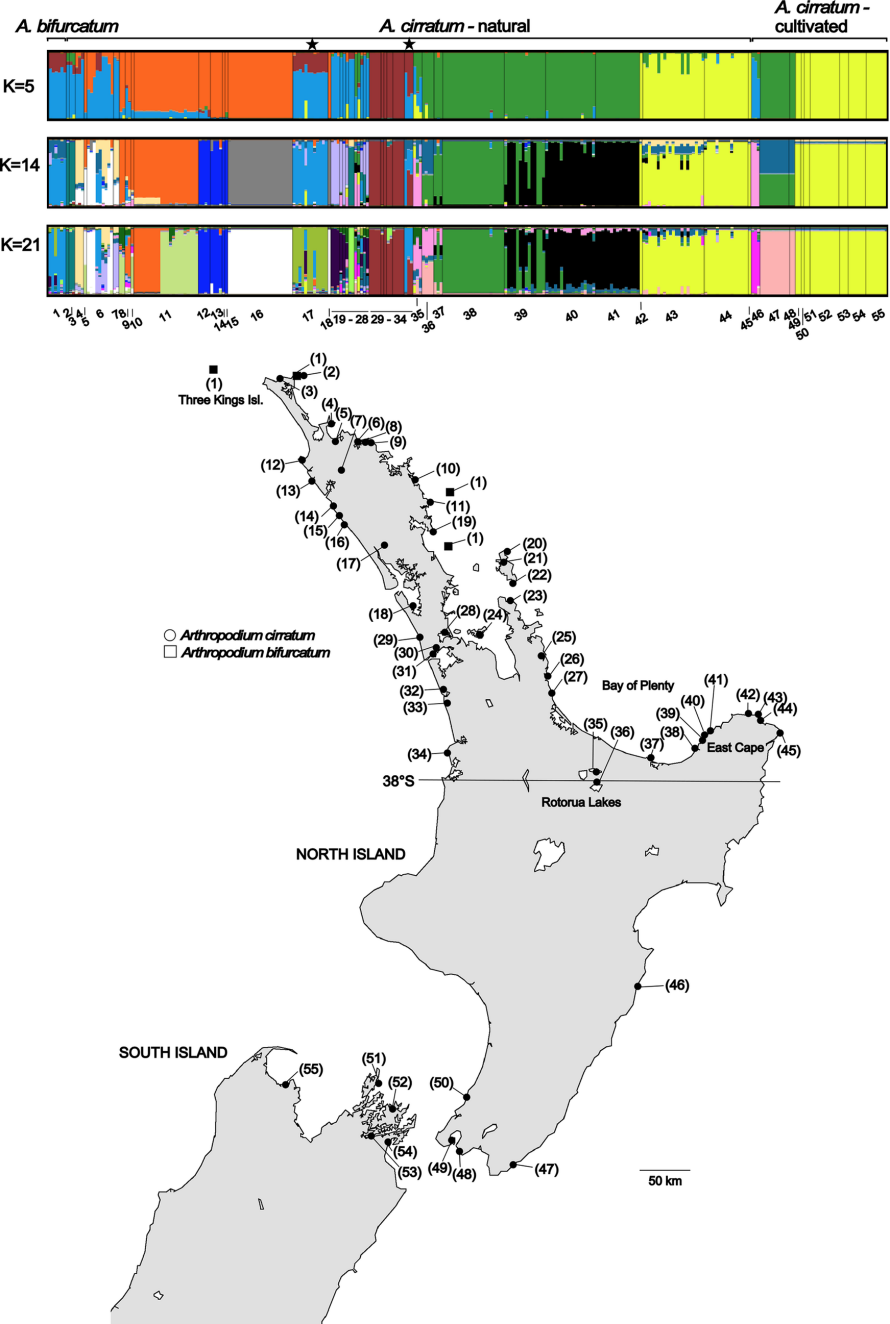
Structure outputs based on microsatellite data from 10 loci. K = 5 was selected as the most likely K value by Structure Harvester but higher levels of K revealed further population subdivision. Outputs for K = 14 and K = 21, which were identified as secondary optima by Structure Harvester are also shown. Individuals are grouped by sampling location and the numbers along the bottom indicate population identification numbers. The two populations marked by stars above the K = 5 plot had the chloroplast haplotype found in *A*. *bifurcatum*. The map shows the distribution of sampling sites. *Arthropodium bifurcatum* sites are identified by (1). For *A*. *cirratum* numbers in brackets are the sites’ identifying number; see Table 1 for more details. For *A*. *cirratum* sites 2–45 are believed to be naturally occurring and sites 46–55 derive from translocations. The position of 38°S, an important biogeographic boundary in New Zealand, is indicated. The basemap was supplied by Kahuroa.

*Arthropodium cirratum* (G.Forst) R.Br. (rengarenga, repihina-papa, maikaika, New Zealand rock lily) (Asparagaceae) is a perennial lily-like herb endemic to New Zealand. It is primarily a coastal species and its natural distribution is thought to be north of around 38°S (Fig 1) [5]. The fleshy roots of *A*. *cirratum* were used as a food source by Māori [6] and its distribution south of 38°S, which is often associated with Māori archeological sites, has been suggested to result from translocations as part of its cultivation [5,7].

**Table 1:** Genetic diversity in *Arthropodium bifurcatum* and *A*. *cirratum* based on ten microsatellite markers.

Species	Location	Latitude, Longitude (°S, °E)	CP haplotype	N	A	P	H_O_	H_E_	F_IS_
*A*. *bifurcatum*	1. Multiple Northland sites (see Fig 1): Three Kings Islands, Surville Cliffs, Poor Knights Islands, Hen Island	-34.1575, 172.1387; -34.3920, 173.0188; -35.4688, 174.7365; -35.8917, 174.7274	A	6	23	3	0.15±0.05	0.22±0.06	0.28±0.13
*A*. *cirratum* (natural populations)	2. Surville Cliffs, Northland	-34.3956, 173.0124	Y	1	12	0	0.20±0.13	-	-
	3. Te Paki, Northland	-34.3956, 173.0124	P	2	16	0	0.30±0.11	-	-
	4. Whatawhiwhi, Northland	-34.8827, 173.3992	V	3	19	3	0.40±0.11	-	-
	5. Taipa, Northland	-34.9895, 173.4727	D	1	10	0	0.00±0.00	-	-
	6. Ranfurly Bay, Northland	-35.0169, 173.7238	W, Z	9	40	3	0.38±0.07	0.55±0.07	0.27±0.11
	7. Mangataipa, Northland	-35.2482, 173.5351	S	2	13	0	0.15±0.11	-	-
	8. Tauranga Bay, Northland	-35.0095, 173.7801	H	2	13	1	0.20±0.13	-	-
	9. Mahinepua, Northland	-34.9974, 173.8491	D	2	13	0	0.15±0.11	-	-
	10. Whangaruru, Northland	-34.3744, 174.3705	O	1	14	0	0.40±0.16	-	-
	11. Matapouri Bay, Northland	-35.5623, 174.5094	O	22	17	2	0.06±0.02	0.25±0.05	0.38±0.13
	12. Ahipara, Northland	-35.1866, 173.1224	L	4	16	1	0.20±0.07	-	-
	13. Whangape, Northland	-35.3651, 173.2187	AD	4	15	1	0.18±0.11	-	-
	14. Waimamaku River, Northland	-35.5843, 173.4179	Q	1	10	0	0.00±0.00	-	-
	15. Waipoua River, Northland	-35.6351, 173.5063	Q	1	10	0	0.00±0.00		
	16. Maunganui Bluff, Northland	-35.7783, 173.5703	I	22	11	1	0.01±0.01	0.00±0.00	-0.23±0.01
	17. Maungaraho, Northland	-36.0205, 173.9727	A	12	28	1	0.44±0.11	0.38±0.08	-0.11±0.12
	18. Shelly Bay, Auckland	-36.5713, 174.3730	I	1	10	0	0.00±0.00		
	19. Bream Head, Northland	-35.8510, 174.5821	G	3	17	1	0.27±0.11	-	-
	20. Needle Rocks, Great Barrier Island	-36.2378, 175.4845	R	1	13	0	0.30±0.15	-	-
	21. Memory Is, Great Barrier Island	-36.2595, 175.4896	R	1	11	0	0.10±0.10	-	-
	22. Raupuke Pt, Great Barrier Island	-36.0012, 175.3588	X	1	9	0	0.10±0.10	-	-
	23. Sandy Bay, Coromandel	-36.5281, 175.4624	F	2	11	0	0.05±0.05	-	-
	24. Waihoka, Waiheke Island	-36.8425, 175.1366	C	1	15	0	0.50±0.17	-	-
	25. Te Huruhuru Pt, Coromandel	-37.0047, 175.8676	C	1	15	0	0.50±0.17	-	-
	26. Whangamata, Coromandel	-37.2200, 175.8888	K	1	14	0	0.40±0.16	-	-
	27. Waihi Beach, Coromandel	-37.3896, 175.9368	K	1	13	1	0.30±0.15	-	-
	28. Waitemata, Auckland	-36.8225, 174.7032	T	1	13	0	0.30±0.15	-	-
	29. Te Henga, Auckland	-36.8924, 174.4437	E	4	12	0	0.13±0.09	-	-
	30. Waitakere, Auckland	-36.9544, 174.6035	E	1	11	0	0.10±0.10	-	-
	31. Orpheus Bay, Auckland	-37.0059, 174.5732	U	1	11	0	0.10±0.05	-	-
	32. Maioro, Waikato	-37.3366, 174.6848	E	2	14	2	0.20±0.11	-	-
	33. Limestone Downs, Waikato	-37.4575, 174.7463	E	4	12	1	0.10±0.08	-	-
	34. Papanui Pt, Waikato	-37.8898, 174.7636	A	3	16	0	0.30±0.09	-	-
	35. Lake Rotoehu, Bay of Plenty	-38.0323, 176.5156	M	3	18	2	0.23±0.09	-	-
	36. Lake Okataina, Bay of Plenty	-38.1385, 176.4019	J	4	11	0	0.00±0.00	-	-
	37. Tauwhare Pa, Bay of Plenty	-37.9830, 177.0691	B	3	12	0	0.03±0.03	-	-
	38. Motu, East Cape	-37.8679, 177.6085	B	21	12	0	0.01±0.01	0.01±0.01	-0.02±0.00
	39. Haparapara, East Cape	-37.7929, 177.6679	J	14	15	0	0.06±0.03	0.11±0.06	0.35±0.14
	40. Waikawa, East Cape	-37 6783, 177.7483	AB	17	17	0	0.11±0.05	0.14±0.06	0.12±0.11
	41. Whanarua, East Cape	-37.6762, 177.7867	AC	15	13	0	0.03±0.03	0.07±0.06	0.57±0.03
	42. Tohora Pirau, East Cape	-37.5515, 178.1589	AC	1	10	0	0.00±0.00	-	-
	43. Hicks Bay, East Cape	-37.5683, 178.2866	C	21	14	0	0.05±0.03	0.10±0.05	0.45±0.14
	44. Onepoto, East Cape	-37.5922, 178.2931	C	15	13	0	0.11±0.09	0.08±0.05	-0.08±0.20
	45. Otiki, East Cape	-37.6870, 178.5423	C	1	10	0	0.00±0.00	-	-
	Total natural *A*. *cirratum*			233	87	20	0.12±0.01	0.60±0.07	0.72±0.09
*A*. *cirratum* (cultivated populations)	**46. Kairakau, Hawke’s Bay**	-39.9333, 176.9333	B	3	16	0	0.27±0.08	-	-
	**47. Tora, Wairarapa**	-41.5088, 175.5060	B	10	10	0	0.00±0.00	0.00±0.00	n/a
	**48. Wainuiomata, Wellington**	-41.4075, 174.8883	B	2	10	0	0.00±0.00	-	-
	**49. Miramar, Wellington**	-41.2968, 174.8258	C	2	10	0	0.00±0.00	-	-
	**50. Paekakariki, Wellington**	-40.9900, 174.9520	C	1	10	0	0.00±0.00	-	-
	**51. Puangiangi Island, Marlborough Sounds**	-40.7663, 173.9812	C	2	10	0	0.00±0.00	-	-
	**52. Titirangi, Marlborough Sounds**	-41.0194, 174.1338	C	10	10	0	0.00±0.00	0.00±0.00	n/a
	**53. Picton, Marlborough Sounds**	-41.2684, 174.0265	C	3	10	0	0.00±0.00	-	-
	**54. Ocean Bay, Marlborough Sounds**	-41.3319, 174.1011	C	6	10	0	0.00±0.00	0.00±0.00	n/a
	**55. Abel Tasman, Golden Bay**	-40.8057, 172.9532	C	7	10	0	0.00±0.00	0.00±0.00	n/a
	Total cultivated *A*. *cirratum*			46	21	0	0.17±0.01	0.25±0.06	0.88±0.06
Total for all *A*. *cirratum*				279	87	-	0.07±0.01	0.43±0.06	0.79±0.06

CP haplotype refers to the chloroplast haplotypes recorded in [8]. N = number of samples, A = number of alleles, P = number of private alleles, Ho and He = observed and expected heterozygosity, respectively. Fis = inbreeding coefficient. He and Fis are only reported for populations with n>5. Location numbers match those on Fig 1. Locations in bold are those believed to derive from Māori translocation.

A recent phylogeographic study using chloroplast sequences provided support that *A*. *cirratum* had been translocated [8]. Within the putative natural range of this species there was a very high level of genetic structuring, with many populations fixed for unique chloroplast haplotypes. This was suggested to result from limited dispersal of the seeds [8]. In contrast, only two out of a total of 29 chloroplast haplotypes were detected from the putative cultivated populations. The geographic distributions of these two haplotypes did not overlap in either their natural or cultivated ranges. Shepherd et al. [8] suggested that these either represent separate introductions from different genetically-distinct source populations within the Bay of Plenty/East Coast region or a single introduction from an unsampled population containing both haplotypes. The cultivation of *A*. *cirratum*, like that of many other pre-European crops grown by Māori, likely stopped in the late 18^th^ century with the introduction of higher-yielding crops by Europeans settlers [7].

A second endemic species of *Arthropodium*, *A*. *bifurcatum*, is restricted to coastal areas in the northern North Island [5]. Although it is most common on islands, *A*. *bifurcatum* is also known from several mainland sites. Based on its ecological associations, *A*. *bifurcatum* is suspected to have declined from the New Zealand mainland following the loss of the seabird-driven vegetation that is a feature of predator-free offshore islands [5]. *Arthropodium bifurcatum* and *A*. *cirratum* are sympatric at a number of locations but no hybrids between them have been observed [5]. Shepherd et al. [8] found that all sampled *A*. *bifurcatum* exhibited a single chloroplast haplotype. This haplotype was also fixed in two populations with the morphology of *A*. *cirratum* (populations 17 and 34; Fig 1), neither of which grows in close proximity to *A*. *bifurcatum*. This haplotype sharing was suggested to result from either an ancient hybridization event or lineage sorting [8].

The chloroplast study of *A*. *cirratum* [8] raised several questions whose investigation requires nuclear DNA data. The chloroplast and nuclear genomes can differ in their mode of transmission, which may result in differing levels of gene flow [9] and thus genetic structuring patterns. Here we use nuclear microsatellite markers to examine the following questions:

1. How many times was *A*. *cirratum* brought into cultivation and how much genetic diversity was lost through the domestication bottleneck?
2. Does the nuclear genome of *A*. *cirratum* have similarly high genetic structuring to that found for the chloroplast genome?
3. What is the relationship between *A*. *cirratum* and *A*. *bifurcatum*, particularly the two *A*. *cirratum* populations with *A*. *bifurcatum* chloroplast genomes?

## Materials and methods

Sampling included the specimens for which chloroplast loci had been sequenced [8], with additional samples for some populations. In total 279 *A*. *cirratum* samples from 54 populations were included with 1 to 22 individuals per population sampled (Table 1). Many populations were small so large numbers of individuals could not be sampled for these. Six samples of *A*. *bifurcatum*, from four sites, were also included; the samples of *A*. *bifurcatum* were pooled as one group for all analyses. Approval to collect samples from conservation land was provided by the Department of Conservation (permits WA-23814-FLO, BOP-23814-FLO, TT-23661-FLO and NO-233360-FLO). Permission to collect samples from the Otari Wilton's Bush botanic gardens was provided under permit 145 and the permission of individual land owners was obtained prior to the collection of samples from private land.

For samples that did not already have DNA available, a modified CTAB method was used to extract genomic DNA (steps 1, 3–7 from Table 1 in [10]. Twelve microsatellite markers developed for *A*. *cirratum* [11] were genotyped. Fluorescent labelling, genotyping and allele scoring followed [11]. Possible scoring errors caused by null alleles, stutter and allelic dropout were assessed with Microchecker v2.2.3 [12].

Matching allele profiles between different individuals were detected using GenAlEx 6.5 [13]. Descriptive statistics including observed (H_O_) and expected heterozygosity (H_E_), number of alleles (A), number of private alleles (P_A_) and inbreeding coefficient (F_IS_) were calculated in GenAlEx 6.5 [13].

Loci were tested for deviation from Hardy–Weinberg equilibrium (HWE) with GenAlEx 6.5. Fisher's exact tests of linkage disequilibrium between loci were calculated in Genepop v.4.2 [14]. The Bonferroni correction was used to correct significance values for multiple tests [15]. Genetic differentiation was estimated by calculating pairwise F_ST_ values between all populations with more than five samples with Arlequin v3.5.2.2 [16]. Global F_ST_ for all *A*. *cirratum* populations was also calculated with Arlequin. For both global and pairwise F_ST_ values, statistical significance was tested by 10 000 permutations whereby for each permutation individuals were randomly exchanged between populations and a new F_ST_ calculated.

To test for correlations between F_ST_ and geographic distance, we performed Mantel tests between the pairwise F_ST_ values and spatial distance among the nine natural populations with more than five samples in Arlequin v3.5.2.2. Both straight-line distances and minimum coastline distances were tested and significance tested with 1000 permutations. Straight-line geographic distances between populations were calculated using the Geographic Distance Matrix Generator [17] and coastal distances were measured from a map.

Population structure was examined with STRUCTURE v2.3.4 [18,19], without prior grouping assumptions. The number of genetic clusters (K) was set between 1 and 25, with 10 permutations for each. We used the admixture model with correlated allele frequencies and ran 100,000 generations of burn-in followed by 500,000 Markov Chain Monte Carlo (MCMC) iterations. The optimal number of genetic clusters (*K*) was obtained by calculating the *Δ*K statistic [20] in STRUCTURE HARVESTER web v.0.6.94 [21]. CLUMPP v.1.1.2 [22] was used to average iterative runs of K and the results visualized graphically with Distruct 1.1 [23].

## Results

### Microsatellite variability in *A*. *cirratum* and A. *bifurcatum*

In some populations, including natural populations demonstrating variation at other loci, all of the individuals at two of the 12 loci, ArtCir18 and ArtCir38, had fixed heterozygotes. These two loci were excluded from further analyses. Microchecker found no evidence of large allele dropout or stuttering. Possible null alleles were inferred for seven loci, each in a single population. Three of these loci were in the Matapouri Bay population (population 11), which has within-population sub-structuring (see Structure analyses below), likely explaining this result. For the remaining four loci the null allele frequencies was estimated to be low (<0.2) and, because previous research has shown that low frequency null alleles have little influence on the detection of genetic differentiation [24], we retained these loci.

Within *A*. *cirratum* the 10 loci displayed a total of 87 alleles, with 4 to 20 alleles per locus (mean ± SD: 8.7 ± 4.6). The six *A*. *bifurcatum* samples had 23 alleles, with 1 to 4 alleles per locus (mean ± SD: 2.3 ± 1.1). *Arthropodium bifurcatum* had three private alleles. Within *A*. *cirratum*, 12 populations had private alleles with one to three private alleles in each (Table 1).

Following sequential Bonferroni correction, no significant linkage disequilibrium was detected among paired loci comparisons. Significant deviation from HWE was observed for two loci, following sequential Bonferroni correction (ArtCir22 in five populations, including the Matapouri Bay population, and ArtCir 26 in two populations).

The mean F_IS_ for *Arthropodium cirratum* was high and positive (F_IS_ = 0.72±0.09), indicating inbreeding. At the population level, the F_IS_ values varied from -0.23 to 0.57 (Table 1). The mean F_IS_ for *A*. *bifurcatum* was 0.28±0.13.

The cultivated populations (populations 46–55) had very low genetic diversity compared to the natural populations. Across the 10 loci only 21 of the 87 *A*. *cirratum* alleles were found in the cultivated populations; all of these were also detected in the natural populations. The cultivated populations have retained only 41.6% of the genetic diversity of the natural populations, as calculated from H_E_.

Genotype matching revealed that all individuals sampled from the cultivated Tora and Wainuiomata populations (47 and 48) shared an identical genotype but this genotype was not detected from any individuals from natural populations. All the alleles found in the Tora and Wainuiomata populations were also found in the natural populations at Haparapara and Motu (populations 38 and 39), except for the fixed allele at locus ArtCir43.

All individuals from the cultivated Miramar and Paekakariki populations (populations 49 and 50) plus all of the South Island individuals (populations 51–55) had an identical genotype and this matched that found in all individuals from the natural populations at Tohora Pirau (42) and Otiki (45) and four individuals from the Hick’s Bay population (43).

The only cultivated population that was not monomorphic across the 10 loci was Kairakau (population 46), whose three individuals showed variation at six loci. None of the three individuals from Kairakau matched any other individuals sampled across all loci.

## Population structuring

Global population differentiation for *A*. *cirratum* was very high (F_ST_ = 0.84). Pairwise F_ST_ values for *A*. *cirratum*, calculated for populations with >5 individuals, were generally high and significant (Table 2). The only non-significant F_ST_ values were between the adjacent populations of Whanarua and Waikawa (populations 40 and 41) and the comparisons between the three cultivated South Island populations (populations 52, 54 and 55). Pairwise F_ST_ values between *A*. *bifurcatum* and the *A*. *cirratum* populations were also high and ranged from 0.14 to 0.93 (Table 2).

**Table 2:** Pairwise F^ST^ values for *Arthropodium cirratum* populations with >5 individuals and *A*. *bifurcatum*.

	*A*. *bifurcatum*	Ranfurly Bay	Matapouri Bay	Maunganui Bluff	Maungaraho	Motu	Haparapara	Waikawa	Whanarua	Hick’s Bay	Onepoto	Tora	Titirangi	Ocean Bay	Abel Tasman
*A*. *bifurcatum* (1)	0.00														
Ranfurly Bay (6)	**0.33**	**0.00**													
Matapouri Bay (11)	**0.75**	**0.49**	0.00												
Maunganui Bluff (16)	**0.93**	**0.66**	**0.82**	0.00											
Maungaraho (17)	**0.14**	**0.30**	**0.64**	**0.82**	0.00										
Motu (38)	**0.93**	**0.77**	**0.88**	**0.99**	**0.81**	0.00									
Haparapara (39)	**0.82**	**0.64**	**0.80**	**0.93**	**0.70**	**0.42**	0.00								
Waikawa (40)	**0.81**	**0.64**	**0.79**	**0.92**	**0.69**	**0.62**	**0.15**	0.00							
Whanarua (41)	**0.86**	**0.68**	**0.83**	**0.96**	**0.73**	**0.76**	**0.26**	0.06	0.00						
Hick’s Bay (43)	**0.84**	**0.70**	**0.82**	**0.94**	**0.72**	**0.89**	**0.77**	**0.73**	**0.78**	0.00					
Onepoto (44)	**0.82**	**0.66**	**0.82**	**0.95**	**0.67**	**0.93**	**0.82**	**0.79**	**0.84**	**0.40**	0.00				
Tora (47)	**0.90**	**0.68**	**0.85**	**0.99**	**0.75**	**0.94**	**0.66**	**0.70**	**0.83**	**0.84**	**0.91**	0.00			
Titirangi (52)	**0.88**	**0.68**	**0.84**	**1.00**	**0.71**	**0.99**	**0.88**	**0.84**	**0.92**	**0.40**	**0.29**	**1.00**	0.00		
Ocean Bay (54)	**0.85**	**0.62**	**0.82**	**0.99**	**0.66**	**0.99**	**0.86**	**0.82**	**0.90**	**0.36**	**0.24**	**1.00**	0.00	0.00	
Abel Tasman (55)	**0.86**	**0.64**	**0.83**	**1.00**	**0.67**	**0.99**	**0.86**	**0.82**	**0.90**	**0.37**	**0.25**	**1.00**	0.00	0.00	0.00

Numbers in brackets are site identification numbers. Significant values are show in bold (P<0.01).

The Structure Harvester analysis of ΔK indicated that the optimal K was 5 (Fig 1, S1 Fig), with secondary optima at K = 3, K = 14 and K = 21 (S1 Fig). At K = 5 most individuals clustered with high probability to a single cluster. At this value of K *A*. *bifurcatum* did not form a distinct cluster but grouped with a number of *A*. *cirratum* populations (populations 2–4, 6–7, 17, 19–23, 26–28 and 46), including the Maungaraho population, which exhibited the *A*. *bifurcatum* chloroplast haplotype. The other *A*. *cirratum* population shown to have an *A*. *bifurcatum* chloroplast haplotype, Papanui Point (population 34), showed affinities to both the cluster containing *A*. *bifurcatum* and also to a second cluster that included populations in close geographic proximity (populations 29–33).

At K = 5 the cultivated populations were assigned to three clusters. The population at Kairakau (46) clustered with *A*. *bifurcatum* and a number of other *A*. *cirratum* populations (see above). The Tora and Wainuiomata populations (47 and 48) clustered with populations from the eastern Bay of Plenty (36–41). The Miramar (49), Paekakariki (50) and South Island populations (51–55) formed a cluster with samples from East Cape (42–45).

At higher values of K additional population structuring was evident (Fig 1), with many populations assigned to single clusters with high probability. One population, at Matapouri Bay (population 11), was assigned with high probability to two distinct clusters at values of K≥18 (see K = 21, Fig 1). Individuals in these two clusters were fixed for a different allele at ArtCir22. Samples at Matapouri Bay were collected from two adjacent headlands approximately 200m apart, separated by a sandy beach where *A*. *cirratum* was not present. The genetic split detected by STRUCTURE corresponds to this geographic separation.

*Arthropodium bifurcatum* did not form a separate cluster until K = 16. At this value of K the Papanui Point *A*. *cirratum* population (34), which shares a chloroplast haplotype with *A*. *bifurcatum*, was split between the *A*. *bifurcatum* cluster and populations 29–33. Individuals from the second population of *A*. *cirratum* with an *A*. *bifurcatum* chloroplast haplotype, the Maungaraho population (17), were assigned with high probability to the *A*. *bifurcatum* cluster until K ≥ 16, when they formed their own cluster.

At K = 14 and K = 21 the cultivated populations 49–55 clustered with those from East Cape (42–45). At K = 14 the Kairakau population (46) grouped with Lake Rotoehu (35) but it formed an independent cluster at K = 21. At K = 14 the Tora (47) and Wainuiomata populations (48) were split between clustering with the Lake Okataina population (36) and a second cluster that included samples from Motu (38), Tauwhare Pa (37) and some individuals from Haparapara (39). At K = 21 the Tora and Wainuiomata individuals were assigned with high probability to a distinct cluster, with individuals from Lake Okataina (36) also showing some affinity to this cluster.

A Mantel test indicated that there was a moderate significant correlation between straight-line geographic and genetic distance (r = 0.421; P<0.005) for the natural populations with more than 5 individuals. This relationship weakened slightly when coastal distances were used (r = 0.396; P<0.02)

## Discussion

### Genetic structuring in *A*. *cirratum*

*Arthropodium cirratum* exhibited very strong genetic differentiation between populations within its natural distribution, and some of this structure can be explained by isolation-by-distance. Population structuring was even detected within the Matapouri Bay site, where two headlands 200m apart had different subpopulations. The nuclear microsatellite results confirm that the high level of genetic structuring previously reported [8] is not restricted to the chloroplast genome and indicates that both pollen and seed dispersal is highly restricted in this species. It has been suggested that *A*. *cirratum* seeds are dispersed by gravity [25] and/or wind [26]. The species is insect pollinated but also uses delayed autonomous self-pollination, where selfing occurs once the chance to outcross has passed [27]. This pollination strategy may explain the large variation in F_IS_ values among populations; i.e., some populations have sufficient pollinators for outcrossing, while others do not. It is possible that clonal propagation has contributed to the population structuring and low diversity observed within some *A*. *cirratum* populations. However, since this species tends to form distinct clumps in different crevices on rock bluffs, the observed patterns cannot solely be explained by clonal propagation and our sampling targeted distinct clumps to avoid collecting clones.

The high structuring at nuclear microsatellites and presence of private alleles in many *A*. *cirratum* populations reinforces our previous recommendation [8] that numerous populations need to be conserved to preserve the genetic diversity of this species.

### Origin of the cultivated *A*. *cirratum* populations

The two chloroplast haplotypes previously detected from the cultivated *A*. *cirratum* populations indicated that these populations had been sourced from the Bay of Plenty/East Cape region. However, it was unclear whether there had been multiple introductions from different sites or a single introduction from a single unsampled variable population [8]. The microsatellite data provides additional insight into the origins of the cultivated population and indicates at least three independent pre-European introductions of *A*. *cirratum* into cultivation.

The cultivated samples with chloroplast haplotype C (populations 49–55) had identical microsatellite profiles. This genotype was also found at four locations in the natural populations: Onepoto, Otiki, Tohora Pirau and Hick’s Bay (populations 42–45). The Tohora Pirau sample has a different chloroplast haplotype, AC [8], thus excluding it as the source of the cultivated plants. The remaining sites, which occur across a 30 km stretch of coastline at East Cape, are the most likely source for populations 49–55.

Cultivated samples with chloroplast haplotype B were more variable at the microsatellite loci than those with haplotype C. The population at Kairakau (46), showed variation at six of the ten microsatellite loci, despite only three individuals sampled. This was considerably more variation than that detected from Tauwhare Pa and Motu (populations 37 and 38), the only natural populations with haplotype B. Those populations only showed variation at two and one of the loci, respectively. The variation found at Kairakau may result from an introduction from an unsampled, possibly extinct, genetically variable population in the vicinity of populations 37 and 38. There is little natural coastal vegetation remaining in the Bay of Plenty [28] and it is likely that *A*. *cirratum* was more widespread in this region in the past. Alternatively, the Kairakau population may derive from multiple introductions from different source populations from this region.

The other cultivated populations with haplotype B, Tora (47) and Wainuiomata (48), had identical microsatellite profiles, suggesting a common origin. However, this genotype did not match any of the individuals we sampled from the natural population. STRUCTURE analyses indicated that these cultivated populations are most similar to natural populations 36–41. However, populations 36 and 39–41 do not have haplotype B, excluding them as the source, and populations 37 and 38 are fixed for a different allele at locus ArtCir43 to the Tora and Wainuiomata samples. This suggests the source of Tora and Wainuiomata was an unsampled, possibly extinct, population from the Bay of Plenty, in the vicinity of populations 37 and 38. This source population is unlikely to be the same as the Kairakau population because the Kairakau samples were fixed for different alleles than those from Tora and Wainuiomata at two loci (ArtCir32 and Art43). This also suggests that the Tora and Wainuiomata populations have an independent origin to the plants from Kairakau and are unlikely to derive via stepping-stone dispersal from Kairakau. Overall our results indicate at least three independent translocation events out of the Bay of Plenty/East Coast region and suggest that both chloroplast and nuclear data can provide complementary sources of information.

### Loss of diversity in cultivated *A*. *cirratum*

All of the cultivated *A*. *cirratum* populations, except for Kairakau, were fixed across all microsatellite loci, indicating the loss of diversity through a ‘domestication bottleneck’. Overall only 41.6% of the nuclear diversity detected in the natural *A*. *cirratum* populations was maintained through the domestication bottleneck, as measure by H_E_. This value is low compared with other perennial crops [29], where an average of 91.4% diversity was retained and is also lower than the average genetic diversity retained though domestication bottlenecks by annual crops (59.9% [30]). As pointed out by Shepherd et al. [8] a number of factors likely contribute to the high loss of genetic diversity through the *A*. *cirratum* domestication bottleneck including the high level of population structuring within the natural distribution of *A*. *cirratum*, the narrow area from which cultivated plants were sourced and the physical isolation of the translocated populations from the natural populations, which has prevented gene flow from introducing additional wild diversity into the cultivated populations.

The lack of variation within most of the cultivated populations (populations 47–55) may indicate that *A*. *cirratum* was cultivated vegetatively through the division of plants. Alternatively the observed low diversity in these populations may be a consequence of low diversity in the source populations, selfing or loss of diversity through the domestication bottleneck (see below) and/or population bottlenecks following the cessation of cultivation. Next-generation sequencing methods that allow screening of many more DNA markers may be able to discriminate between these hypotheses.

### *A*. *cirratum* and *A*. *bifurcatum*

Two populations of *A*. *cirratum* had previously been shown to share a chloroplast haplotype with *A*. *bifurcatum*. The nuclear microsatellites also indicate a mixed ancestry for these populations. In the STRUCTURE analyses the Papanui Point population (34) was assigned to two genetic clusters: one containing *A*. *bifurcatum* and the other comprising geographically adjacent *A*. *cirratum* populations. The Maungaraho population (17) clustered with *A*. *bifurcatum* in the STRUCTURE analyses until it formed its own cluster at higher K. Pairwise F_ST_ values also indicated a closer relationship between *A*. *cirratum* from Maungaraho and *A*. *bifurcatum* than any other comparisons (including within *A*. *cirratum*).

There are two possible explanations for the patterns observed: shared ancestral variation and interspecific hybridization, and they are often difficult to distinguish because they produce similar patterns of allele sharing [31]. The small population sizes in *A*. *cirratum* favours a hybridization hypothesis (shared polymorphism is more likely in large effective population sizes [32]). The assignment of the Papanui Point plants to a geographically adjacent *A*. *cirratum* cluster (as well as to the *A*. *bifurcatum* cluster) in STRUCTURE also points to an origin involving hybridization rather than shared ancestral polymorphism. For the observed pattern to have arisen through shared ancestral variation, *A*. *bifurcatum* must have diverged subsequent to the development of the genetic structuring within *A*. *cirratum*. However, the divergent chloroplast haplotype in *A*. *bifurcatum* [8] suggests this is not the case. The fact that the nearest *A*. *bifurcatum* to Papanui Point is over 200 km away could infer a chance long distance dispersal event in *A*. *bifurcatum* or indicate that this species had a wider distribution in the past. Heenan et al. [5] had noted sporadic northern North Island mainland occurrences of *A*. *bifurcatum*. Since that publication our own fieldwork has extended the mainland range of this species, discovering highly fragmented *A*. *bifurcatum* populations–sometimes comprising single plants still occur scattered as far south as Whangamata and Ngatutura Point. Otherwise *A*. *bifurcatum* is a common plant on predator-free offshore islands of northern North Island [5]. In those places it is a feature of the low often scrubby vegetation associated with seabird nests and roosts (i.e. the ‘ornithocoprophilous ecosystem’ [32–34]. As this ecosystem was once present through all of the main islands of New Zealand, it seems likely that the hybridism indicated at Maungaraho Rock and Papanui Point provides further evidence of the historical loss of the flora associated with these seabirds from the mainland.

## Supporting information

S1 Fig Results of implementing the Evanno method for detecting the number of K groups that best fit the *Arthropodium* microsatellite data. According to the ΔK, K = 5 represents the optimal structure partition in our dataset, with secondary optima at K = 14 and K = 21.
(PDF)

S1 Table Genotypes at 12 microsatellite loci for *Arthropodium cirratum* and *A*. *bifurcatum*. (XLSX)

## References

[1] Anderson AJ. The chronology of colonization in New Zealand. Antiquity. 1991; 65: 767–795. 10.1017/S0003598X00080510

[2] Wilmhurst JM, Hunt TL, Lipo CP, Anderson AJ. High-precision radiocarbon dating shows recent and rapid initial human colonization of East Polynesia. Proc Natl Acad Sci USA. 2011; 108: 1815–1820. 10.1073/pnas.1015876108 21187404 PMC3033267

[3] Whistler WA. Plants of the Canoe People.—an ethnobotanical voyage through Polynesia Kaua'i, Hawai'i: National Tropical Botanical Garden; 2009.

[4] Yen D. The achievements of the Maori agriculturalist In: Harris W, Kapoor P, editors. Nga Mahi Maori o Te Wao nui a Tane; contributions to an international workshop on ethnobotany. Christchurch: DSIR Botany Division; 1990.

[5] Heenan PB, Mitchell AD, de Lange PJ. *Arthropodium bifurcatum* (Asparagaceae), a new species from northern New Zealand. NZ J Bot. 2004; 42: 233–246. 10.1080/0028825X.2004.9512900

[6] Colenso W. On the vegetable food of the ancient New Zealanders. Trans R Soc NZ 1880; 13: 3–38.

[7] Harris GF, Te Whaiti H. Rengarenga lilies and Maori occupation at Mātakitaki-a-Kupe (Cape Palliser): An ethnobotanical study. J Polyn Soc. 1996; 105: 271–286.

[8] Shepherd LD, de Lange PJ, Cox S, McLenachan PA, Roskruge NR, Lockhart PJ. Evidence of a strong domestication bottleneck in the recently cultivated New Zealand endemic root crop, *Arthropodium cirratum* (Asparagaceae). PLoSONE. 2016; 11(3): e0152455. 10.1371/journal.pone.0152455 PMC4806853 27011209

[9] Petit RJ, Duminil J, Fineschi S, Hampe A, Salvini D, Vendramin GG. Comparative organization of chloroplast, mitochondrial and nuclear diversity in plant populations. Mol Ecol. 2005; 14: 689–701. 10.1111/j.1365-294X.2004.02410.x 15723661

[10] Shepherd LD, McLay TGB. Two micro-scale protocols for the isolation of DNA from polysaccharide-rich plant tissue. J Plant Res. 2011; 124: 311–314. 10.1007/s10265-010-0379-5 20927638

[11] Bulgarella M, Biggs PJ, de Lange PJ, Shepherd LD. Isolation and characterization of microsatellite loci from *Arthropodium cirratum* (Asparagaceae). Appl Plant Sci. 2017; 5(8) 10.3732/apps.1700041 28924514 PMC5584818

[12] Van Oosterhout C, Hutchinson WF, Willis DPM, Shipley P. MICROCHECKER: software for identifying and correcting genotyping errors in microsatellite data. Mol. Ecol. Resour. 2004; 18: 535–538.

[13] Peakall R, Smouse PE. GenAlEx 6.5: Genetic analysis in Excel. Population genetic software for teaching and research—An update. Bioinformatics. 2012; 28: 2537–2539. 10.1093/bioinformatics/bts460 22820204 PMC3463245

[14] Rousset F. GENEPOP’007: A complete re-implementation of the GENEPOP software for Windows and Linux. Mol. Ecol. Resour. 2008; 8: 103–106. 10.1111/j.1471-8286.2007.01931.x 21585727

[15] Holm S (1979) A simple sequential rejective method procedure. Scand J Stat. 1979; 6: 65–70.

[16] Excoffier L, Lischer HEL. Arlequin Suite Ver 3.5: A new series of programs to perform population genetics analyses under Linux and Windows. Mol. Ecol. Resour. 2010;10:564–567. 10.1111/j.1755-0998.2010.02847.x 21565059

[17] Ersts PJ. Geographic Distance Matrix Generator (version 1.2.3) (2015) American Museum of Natural History, Center for Biodiversity and Conservation Available: http://biodiversityinformatics.amnh.org/open_source/gdmg

[18] Pritchard JC, Stephens M, Donnelly P. Inference of population structure using multilocus genotype data. Genetics. 2000; 155: 945–959. 10835412 10.1093/genetics/155.2.945 PMC1461096

[19] Falush D, Stephens M, Pritchard JK. Inference of population structure using multilocus genotype data: Dominant markers and null alleles. Mol Ecol Notes. 2007; 7: 574–578. 10.1111/j.1471-8286.2007.01758.x 18784791 PMC1974779

[20] Evanno G, Regnaut S, Goudet J. Detecting the number of clusters of individuals using the soft ware STRUCTURE: A simulation study. Mol Ecol. 2005; 14:2611–2620. 10.1111/j.1365-294X.2005.02553.x 15969739

[21] Earl DA, vonHoldt BM. STRUCTURE HARVESTER: A website and program for visualizing STRUCTURE output and implementing the Evanno method. Conserv Genet Resour. 2012; 4: 359–361.

[22] Jakobsson M, Rosenberg NA. CLUMPP: a cluster matching and permutation program for dealing with label switching and multimodality in analysis of population structure. Bioinformatics. 2007; 23:1801–1806. 10.1093/bioinformatics/btm233 17485429

[23] Rosenberg NA. DISTRUCT: a program for the graphical display of population structure. Mol. Ecol. Resour. 2004; 4:137–138.

[24] Carlsson J Effects of microsatellite null alleles on assignment testing. J Hered. 2008; 99: 616–623. 10.1093/jhered/esn048 18535000

[25] Reid I, Sawyer J, Rolfe J. Introduction to the plant life in New Zealand: Plant conservation training module 1 The New Zealand Plant Conservation Network 2009; Available: http://nzpcn.org.nz/publications/Module_1_full_document.pdf

[26] Thorsen MJ, Dickinson KJM, Seddon PJ. Seed dispersal systems in the New Zealand flora. Perspect Plant Ecol Evol Syst. 2009; 11: 285–309. 10.1016/j.ppees.2009.06.001

[27] Zhou W, Li D-Z, Wang H. Coexistence of delayed autonomous self-pollination and deceptive pollination in *Arthropodium cirratum* (Asparagaceae). Plant Diversity Resour. 2012; 34: 187–191. 10.3724/SP.J.1143.2012.11169

[28] Department of Conservation. Conservation management strategy for Bay of Plenty Conservancy, 1997–2007 1996; Volumes 1 & 2 Department of Conservation, Rotorua.

[29] Gross BL, Henk AD, Richards CM, Fazio G, Volk GM. Genetic diversity in *Malus ×domestica* (Rosaceae) through time in response to domestication. Am J Bot. 2014; 101: 1770–1779. 10.3732/ajb.1400297 25326619

[30] Miller AJ, Gross BL. From forest to field: perennial fruit crop domestication. Am J Bot. 2011; 98: 1389–1414. 10.3732/ajb.1000522 21865506

[31] Muir G, Schlötterer C. Evidence for shared ancestral polymorphism rather than recurrent gene flow at microsatellite loci differentiating two hybridizing oaks (*Quercus* spp.). Mol Ecol. 2005; 14: 549–561. 10.1111/j.1365-294X.2004.02418.x 15660945

[32] Pamilo P, Nei M. Relationships between gene trees and species trees. Mol Biol Evol. 1998; 5: 568–583. 10.1093/oxfordjournals.molbev.a040517 3193878

[33] Ornduff R. Ornithocoprophilous endemism in Pacific Basin angiosperms. Ecology. 1965; 46: 846‒847.

[34] Norton DA, de Lange PJ, Garnock-Jones PJ, Given DR. The role of seabirds and seals in the survival of coastal plants: lessons from New Zealand *Lepidium* (Brassicaceae). Biodivers Conserv. 1997; 6: 765‒785.

