# Supplementary figures and images for: Genetic structuring of the coastal herb Arthropodium cirratum (Asparagaceae) is shaped by low gene flow, hybridization and prehistoric translocation
Source: PLoS One. 2018 Oct 17;13(10):e0204943. doi: 10.1371/journal.pone.0204943 (PMC6192600; doi:10.1371/journal.pone.0204943)

$$\text{DeltaK} = \text{mean}(|L''(K)|) / \text{sd}(L(K))$$

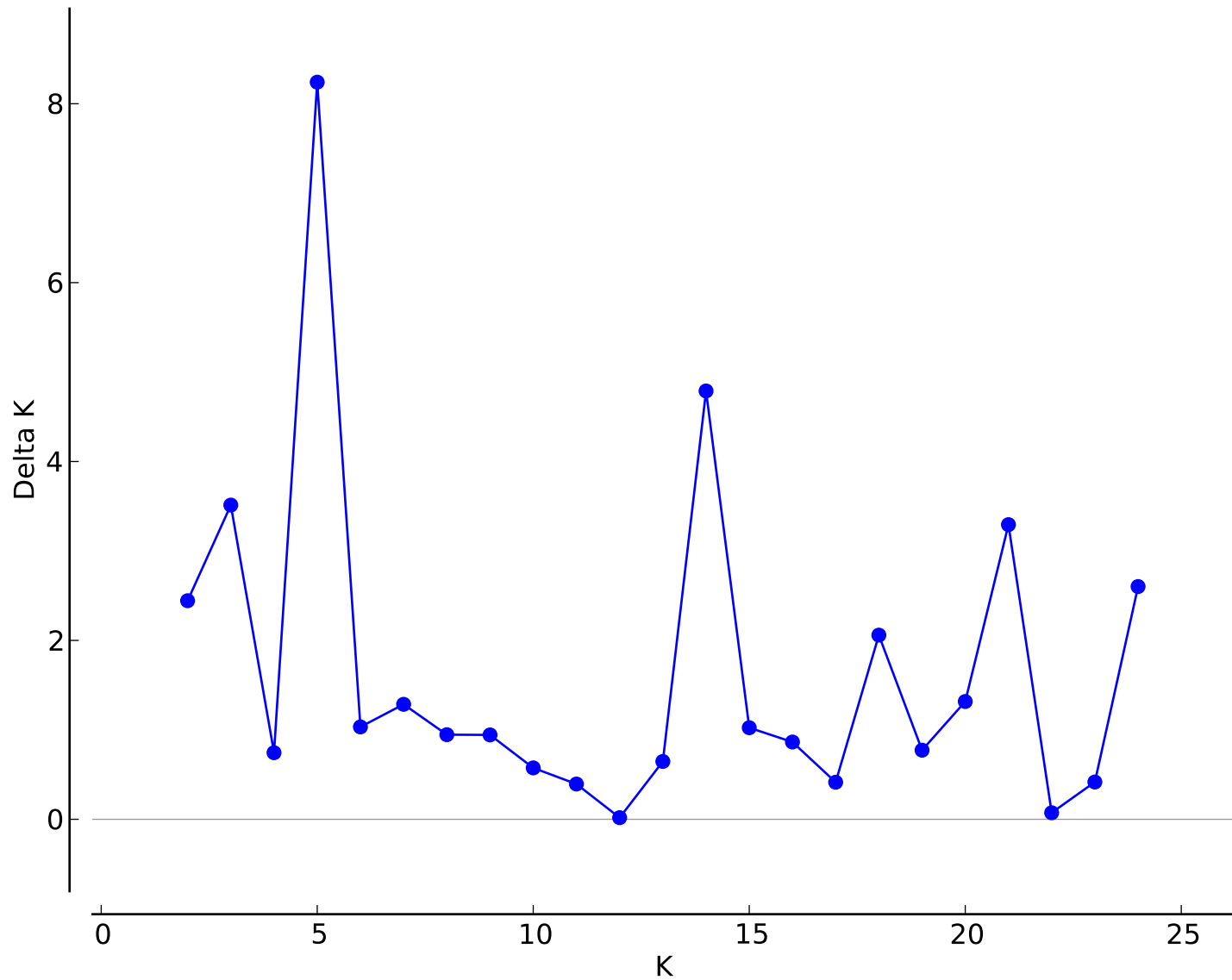

Supplement: S1 Fig — According to the ΔK, K = 5 represents the optimal structure partition in our dataset, with secondary optima at K = 14 and K = 21. (PDF) [file pone.0204943.s001.pdf]
